# Detecting anomalies in smart wearables for hypertension: a deep learning mechanism

**DOI:** 10.3389/fpubh.2024.1426168

**Published:** 2025-01-15

**Authors:** C. Kishor Kumar Reddy, Vijaya Sindhoori Kaza, R. Madana Mohana, Mohammed Alhameed, Fathe Jeribi, Shadab Alam, Mohammed Shuaib

**Affiliations:** ^1^Stanley College of Engineering and Technology for Women, Hyderabad, India; ^2^Department of Artificial Intelligence and Data Science, Chaithanya Bharathi Institute of Technology, Hyderabad, Telangana, India; ^3^Department of Computer Science, College of Engineering and Computer Science, Jazan University, Jazan, Saudi Arabia

**Keywords:** deep learning, machine learning, smart health monitoring, smart wearables, hypertension

## Abstract

**Introduction:**

The growing demand for real-time, affordable, and accessible healthcare has underscored the need for advanced technologies that can provide timely health monitoring. One such area is predicting arterial blood pressure (BP) using non-invasive methods, which is crucial for managing cardiovascular diseases. This research aims to address the limitations of current healthcare systems, particularly in remote areas, by leveraging deep learning techniques in Smart Health Monitoring (SHM).

**Methods:**

This paper introduces a novel neural network architecture, ResNet-LSTM, to predict BP from physiological signals such as electrocardiogram (ECG) and photoplethysmogram (PPG). The combination of ResNet’s feature extraction capabilities and LSTM’s sequential data processing offers improved prediction accuracy. Comprehensive error analysis was conducted, and the model was validated using Leave-One-Out (LOO) cross-validation and an additional dataset.

**Results:**

The ResNet-LSTM model showed superior performance, particularly with PPG data, achieving a mean absolute error (MAE) of 6.2 mmHg and a root mean square error (RMSE) of 8.9 mmHg for BP prediction. Despite the higher computational cost (~4,375 FLOPs), the improved accuracy and generalization across datasets demonstrate the model’s robustness and suitability for continuous BP monitoring.

**Discussion:**

The results confirm the potential of integrating ResNet-LSTM into SHM for accurate and non-invasive BP prediction. This approach also highlights the need for accurate anomaly detection in continuous monitoring systems, especially for wearable devices. Future work will focus on enhancing cloud-based infrastructures for real-time analysis and refining anomaly detection models to improve patient outcomes.

## 1 Introduction

### 1.1 Smart health monitoring

One of the most significant developments in the healthcare industry in the current digital age is smart health care. Traditional medicine based on bioengineering has started to gradually digitalize information due to scientific theory and technological advancements. The healthcare system continuously monitors a patient by examining a variety of data and extrapolating a positive outcome from previous instances of such continuous monitoring. In Intensive Care Units (ICUs), continuous monitoring of patients is standard practice, allowing healthcare providers to access critical information in real-time. This monitoring can be lifesaving for conditions such as diabetes, asthma attacks, heart failure, and hypertension. Smart medical devices can connect to smartphones, enabling the seamless transmission of important patient data to clinicians. These gadgets also record data on blood pressure, weight, blood sugar, and oxygen levels. Smart health care makes it possible for people from a variety of backgrounds (such as doctors, nurses, caregivers for older family members, and patients) to find suitable information and results, appropriate information, and solutions, reduce medical errors, improve care, and reduce expenses at the right time in the health-care department/facilities (1). Several methods are used in smart health care, together with the usage of devices such as mobiles, computers, and televisions, along with various networks, like wide area networks (WANs), local area networks (LANs), and body area networks (BANs). The parameters that are most frequently tracked include blood heat, heart rate, blood pressure, and motion detection.

This research focuses specifically on arterial blood pressure (BP) monitoring, which plays a critical role in managing conditions like hypertension and cardiovascular diseases. The continuous monitoring provided by smart devices enhances real-time assessments, especially in individuals who may lack awareness of their vital signs or have varying levels of clinical knowledge (2). Smart medical devices, such as wearables, help both patients and healthcare providers to access relevant information. These devices track important metrics like blood pressure, heart rate, and oxygen levels, thus offering more accessible and timely interventions, particularly for those in remote areas or with limited access to healthcare (3). SHM empowers diverse users, from patients to healthcare professionals, by reducing medical errors, improving patient care, and cutting healthcare costs by providing real-time, continuous data transmission through networks like LANs and BANs.

Table 1 presents a sample of commonly used wearable sensor technologies, focusing specifically on their applications related to arterial blood pressure (BP) monitoring and other cardiovascular assessments. While this table does not encompass the full breadth of wearable devices available, it highlights technologies particularly relevant to BP prediction and management, as well as related clinical applications such as arrhythmia detection and heart failure management.

**Table 1:** Summary of wearable sensor technologies and clinical applications.

Sensor technology	Device type	Measurements	Clinical applications
PPG	Smartwatch or Band	Heart Rate Variability (HRV), Heart Rate (HR), Blood pressure (BP) without a cuff, oxygen saturation (SaO2), Heart Rate, Sleep Stages, Pulse-based Rhythm Detection, and Stroke Volume	Prediction of arterial blood pressure; Evaluation of risk in both healthy and cardiovascularly ill individuals; screening for and treatment of hypertension; identification and diagnosis of arrhythmias; tracking of sleep; Management of heart failure
ECG	Smart Ring	both single- and multiple-lead ECGs, ongoing or only when necessary observation, interval assessments (such as QTc), detection of arrhythmias, Changes in electrolyte abnormalities	Prediction of arterial blood pressure; Evaluation of risk in both healthy and CVD individuals; screening for and treatment of hypertension; identification and diagnosis of arrhythmias; diagnosis of acute coronary syndrome; extended QTc diagnosis Management of heart failure
Accelerometer	Chest Strap	Steps taken, force of impact, speed, amount of idle time, and exercise	Monitoring physical activity; Assessing risk in both healthy and CVD-afflicted individuals; Cardiopulmonary telerehabilitation; management of heart failure
Barometer	Wristband	Stair count	Monitoring physical activity; Assessing risk in both healthy and CVD-afflicted individuals; Cardiopulmonary telerehabilitation; management of heart failure
GPS	Smart Clothing	Travel distance and burned calories	Monitoring physical activity; Assessing risk in both healthy and CVD-afflicted individuals; Cardiopulmonary telerehabilitation; management of heart failure
Biometric Sensors	Smart Earbuds	Constant Monitoring of Electrolytes and Blood Glucose	monitoring blood sugar levels continuously; managing heart failure
		Non-invasive electrolyte levels in saliva and sweat and state of hydration	
Biomechanical	Smart Shoes	Ballistocardiograms, Seismocardiograms, Dielectric sensors	Weight, body vibrations, lung fluid volume, stroke volume, and cardiac output

### 1.2 Hypertension: conditions for detecting hypertension

Hypertension is a significant global health concern, affecting millions and contributing to a higher risk of cardiovascular diseases. Blood pressure (BP) is a dynamic physiological measure that fluctuates minute by minute, influenced by various environmental and physiological factors (4). Continuous monitoring of BP helps detect trends that might indicate early signs of hypertension or cardiovascular strain. Home BP monitoring is gaining prominence as it offers valuable insights into BP fluctuations throughout the day and night, potentially unveiling conditions like “white coat” hypertension or irregularities linked to stress. This monitoring in diverse contexts allows for more informed decisions in treatment and management, reducing risks such as heart disease or hypertension-induced mortality. Effective BP control is especially crucial for reducing cardiovascular risks, particularly in older adults. While BP measurement has been shown to be an effective predictor of outcomes in cardiovascular disease, a better understanding of BP levels and variability could enhance risk stratification. It may facilitate the detection of “white coat” hypertension and assess excessive BP responses to various stresses. Variations in BP levels between day and night can also provide important information regarding the cardiovascular system (5). Furthermore, the comorbidity of mental illnesses and hypertension is linked to a higher cardiovascular mortality than hypertension alone, as hypertensive patients are more prone to experience anxiety. Effective BP control can decrease the risk of cardiovascular disease (CVD) and mortality in older individuals. BP varies over both short and long periods, including days, months, quarters, or years (6). Home BP monitoring, highly advised as a supplement to standard BP measurement in recent hypertension guidelines, plays a major role in the management of hypertension.

### 1.3 What are medical anomalies and why are they different?

*Medical anomalies* refer to deviations from typical physiological patterns, which may indicate underlying conditions or pathologies. These deviations can be congenital (present at birth) or acquired over time. For instance, variations in blood pressure (BP) could point to cardiovascular disorders, while other anomalies might suggest arrhythmias or metabolic imbalances.

A critical distinction must be made between anomalies and artifacts in medical data. Anomalies reflect genuine physiological irregularities that could suggest disease or abnormal conditions. In contrast, artifacts are errors or distortions in the data—often resulting from sensor misreading or environmental factors—that do not represent real physiological conditions. For example, sudden fluctuations in BP readings could be caused by movement or sensor misalignment rather than an actual BP variation. The machine learning (ML) system presented in this paper focuses primarily on detecting anomalies—deviations in physiological signals such as BP that may indicate an abnormal health state. However, distinguishing between true anomalies and artifacts is also essential to ensure accuracy in diagnoses. This research integrates signal processing techniques within deep learning models to filter out artifacts and enhance the detection of clinically relevant anomalies. To handle medical imaging tasks like classification and segmentation, anomaly detection is one potential methodology that can make use of semi-supervised and unsupervised methods.

**Figure 1:**
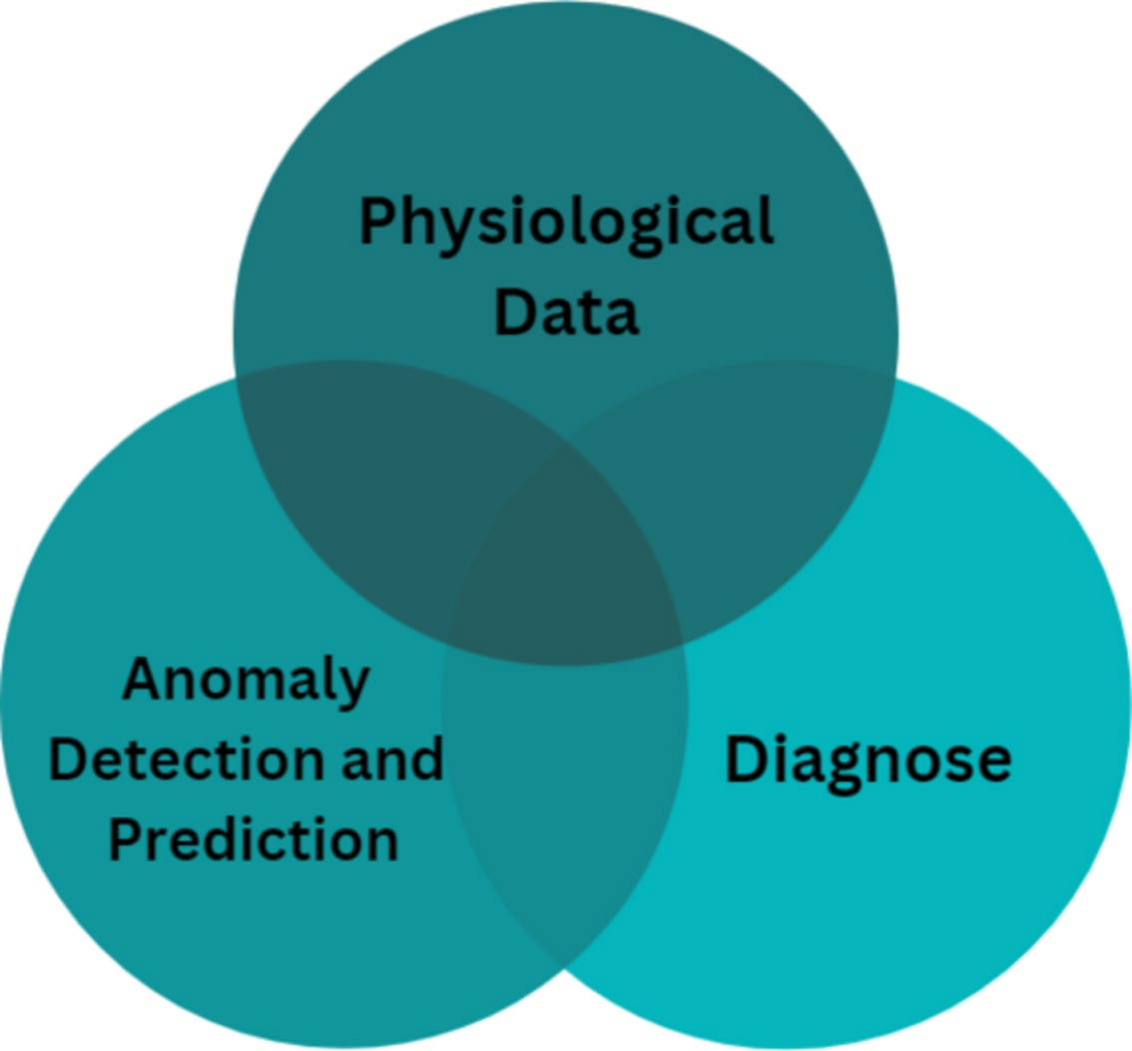
Key phases of processing medical data and their connection to anomaly detection.

Figure 1 illustrates the essential phases of processing medical data using machine learning for anomaly detection. The figure outlines a step-by-step flow from data acquisition to prediction and diagnosis, highlighting how each phase is related to anomaly detection:

- Prediction: Machine learning algorithms predict the future state of physiological signals such as BP, helping clinicians anticipate adverse events or trends (e.g., a gradual increase in BP).
- Diagnosis: By analyzing physiological signals, machine learning models can help identify pathological symptoms (e.g., hypertensive crises or arrhythmias).

These two tasks—prediction and diagnosis—are closely linked to anomaly detection since they enable identification of abnormal patterns in the data. The model extracts distinct features from the data, thereby improving diagnostic accuracy and delivering insights into patient health.

Key challenges in medical anomaly detection include:

- Test sensitivity: High sensitivity is required to detect subtle deviations accurately, ensuring that no abnormality is overlooked during diagnosis.
- Patient-specific factors: An effective model must account for individual differences in physiological baselines, ensuring that anomalies are detected based on personalized norms rather than generalized data.

Given these challenges, medical anomaly detection typically falls under supervised learning, where models are trained on labelled data (normal vs. abnormal) to identify anomalies. This contrasts with other domains, where anomaly detection is often an unsupervised task due to the absence of predefined labels (7).

### 1.4 Why use deep learning for medical anomalies?

Deep learning (DL) has emerged as a potent instrument in biomedical research because of its capacity to handle the intricate problems related to the identification of medical anomalies (8). The key advantages of deep learning, particularly in this context, include:

- Non-linearity modeling: Medical data is often non-linear, with complex relationships between variables. Deep learning models, such as the ResNet-LSTM, are capable of capturing these non-linear relationships, making it easier to distinguish between normal and abnormal physiological states.
- Handling data discrepancies: Medical data often contains inconsistencies or noise, whether due to artifacts or natural variability in patient signals. Deep learning models can manage these discrepancies by learning patterns from large datasets, thereby filtering out irrelevant variations and focusing on clinically significant changes.

**Figure 2:**
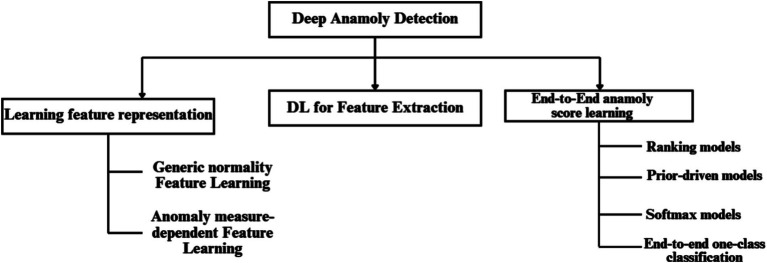
A hierarchical taxonomy of current deep anomaly detection techniques.

In this paper, we leverage a ResNet-LSTM architecture for its ability to model both spatial and temporal features. This enables the model to uncover long-term dependencies in physiological data without the need for explicit feature engineering. Specifically, this approach helps identify BP anomalies by analyzing patterns in photoplethysmogram (PPG) and electrocardiogram (ECG) signals over time. The ResNet component effectively extracts spatial features from the data, while the LSTM component captures temporal relationships, enhancing the model’s predictive power in detecting deviations. By applying this deep learning framework, the model is able to provide continuous, real-time monitoring of physiological signals, making it a robust tool for identifying true anomalies while minimizing the influence of artifacts. Figure 2 provides a hierarchical taxonomy of current deep learning techniques used in anomaly detection, illustrating how different models (including ResNet-LSTM) fit within the broader landscape of anomaly detection approaches.

### 1.5 Objective of paper

This paper’s main objective is to use DL and ML techniques to investigate the transformative potential of SHM. It focuses specifically on the application of neural network architectures, ResNet-LSTM in particular, for the prediction of arterial blood pressure. The aim of this paper is to assess the efficacy of SHM in providing reliable, affordable, and timely health monitoring services, especially in remote areas. The study aims to contribute to the paradigm shift in health data assessment and anomaly detection by integrating intelligent sensors that can monitor health in real-time. It emphasizes the significance of continuous monitoring through wearables.

The first section of the paper introduces the problems that the genesis and transmission of diseases present to the healthcare sector, highlighting the need for creative solutions. The concept of SHM and its potential to transform the assessment of health data is then explored in depth. In the research methodology section, it is explained how physiological signals like PPG and ECG are used to predict arterial blood pressure using deep learning, specifically ResNet-LSTM. A thorough analysis of the ResNet-LSTM network’s performance, including MAE and RMSE values, is provided in the results section. The network’s accuracy across all BP prediction scenarios is demonstrated by numerical values. Interpreting the results, the discussion highlights the importance of accurate anomaly detection and wearables for continuous monitoring. Throughout, the research emphasizes the practical implications of the research in addressing current healthcare challenges and promoting personalized, effective health monitoring solutions.

Table 2 summarizes the types of anomaly detection techniques used in your paper, including deep learning, machine learning, statistical methods, and hybrid approaches. The precise method used, the kind of data analysis, reciprocity, online/offline nature, flexibility, and data processing strategy are given for each technique.

**Table 2:** Summary of anomaly detection techniques.

Technique	Type	Data analysis	Online/Offline	Reciprocity	Adaptability	Data processing
ResNet-LSTM	Deep Learning	Physiological Signals (ECG, PPG)	Online	Temporal	Non-adjustable	Central
WaveNet+LSTM	Machine Learning	Physiological Signals (ECG, PPG)	Offline	Temporal	Non-adjustable	Central
Clustering Algorithm	Statistical Method	Physiological Signals (ECG, PPG)	Offline	Spatial	Non-adjustable	Distributed
Transfer Learning	Hybrid	Image Features	Offline	-	Adjustable	Central

The current methods for detecting anomalies and sensor faults are briefly explained in the following section. The suggested method for detecting sensor anomalies is presented in Section 3. In Section 4, experiments and findings are covered along with a comparison of the suggested strategy with related approaches. Conclusion and potential future work are presented in Sections 5 and 6, respectively.

## 2 Literature survey

Many important factors, such as data processing algorithms, communication networks, sensor selection, contact-based versus contactless techniques, and other design considerations, must be carefully considered to create a dependable remote monitoring system. Numerous review papers offer perceptive evaluations of smart technology by examining it from multiple perspectives.

While Ohta et al. (37) focused on developing a health monitoring system especially for senior citizens who live alone to ease their anxieties and encourage independent living, while Tamura et al. (38) investigated the development of a home health monitoring system that did not forbid activities like bathing, sleeping, or urinating. These studies paved the way for the creation of intelligent wearables for health detection that add new features on a regular basis. Deep learning techniques are utilized by researchers for medical anomaly identification.

Clifford et al. (4) showcased the potential of computational methods in cardiology through their work on the categorization of heart sound recordings as normal or abnormal. In automated rehabilitation, Wang et al. (9) used deep back propagation–LSTM networks for upper-limbs EMG signal categorization. In the context of infectious diseases, Singh et al. (10) created a multi-objective differential evolution-based convolutional neural network for COVID-19 patient classification from chest CT images. Chang et al. (3) demonstrated the precise classification of genetic alterations in gliomas using deep-learning convolutional neural networks for applications other than healthcare.

Using image, audio, and inspection robot sensors, Hea et al. (39) investigated the connection between technology and infrastructure maintenance and developed a non-invasive method for fault diagnosis and detection in water distribution systems. Motwani et al. (1) provided a comprehensive analysis of machine learning-based ubiquitous and intelligent healthcare monitoring frameworks and provided insights into novel and developing treatments for patients with chronic illnesses. Several review articles covering a range of fields discussed anomaly detection. García-Macías and Ubertini (Springer) integrated SHM systems, focusing on data fusion and unsupervised learning to identify damage. Aliyu et al.’s paper, “Anomaly Detection in Wearable Location Trackers for Child Safety,” focused on microprocessors and microsystems. Churová et al. proposed an anomaly detection method for real-world data (11).

The study carried out by Hamieh et al. (12) sheds light on a noteworthy and demanding application of remote monitoring: mental health. They highlight the value of using unsupervised learning to spot relapses in individuals with psychotic disorders, demonstrating the potential benefits of objective, non-intrusive monitoring for early intervention and improved patient outcomes. Further research into AI-powered mental health monitoring systems that safeguard user privacy and provide carers and clinicians with useful information is made possible by this study. Jahan et al. (13) introduce us to a new field by using smartwatch technology for activity recognition within the context of religious rites like salat. This study shows how adaptable remote monitoring can be, going beyond traditional applications in fitness and health to satisfy cultural and religious demands. Think about the benefits that come from tailoring activity detection algorithms to various practices so that individuals can meaningfully monitor their participation and adherence.

The application of remote monitoring in human behavior analysis is elaborated upon by Bozdog et al. (14). Their research demonstrates how anomalies can be detected and intricate human behavior patterns can be deciphered using wearable sensors and machine learning. This opens the door to applications like risk prediction, personalized coaching, and even environmental adaptation based on real-time behavioral data. Our primary concerns as we use these technologies to understand human behavior should be ethics and user privacy. The resource Kalpana et al. (40) was helpful as it gathered an extensive overview of deep learning methods for anomaly identification in human activity recognition, which helped in formulating the proposed model and understand the scope and need for this research work. This provides a comprehensive summary of current research trends and identifies areas that warrant additional research. By highlighting the benefits and drawbacks of various algorithms, this paper lays the foundation for researchers to build on current understanding and push the boundaries of human activity detection accuracy and interpretability. By adding to the body of knowledge, these combined efforts promote the development of remote monitoring systems and increase their efficacy in a range of applications. All the existing works have been summarized for a better understanding in Table 3.

**Table 3:** Summary and insights obtained from existing literature.

Reference	Methods	Techniques	Results	Problems identified
García-Macías and Ubertini (Springer)	Structural health monitoring (SHM) systems	Data fusion, unsupervised learning for damage identification	Incorporation of SHM systems, emphasis on data fusion and unsupervised learning	Customization of activity detection algorithms
Aliyu et al.	Wearable location trackers: detecting anomalies	Microprocessors, microsystems	Centered on wearable location trackers’ anomaly detection for kid safety	Privacy concerns
Churová et al. (2020) (11)	Real-world data anomaly detection technique	Not specified	Proposed real-world data anomaly detection technique	Privacy concerns
Hamieh et al. (2023) (12)	Unsupervised learning for mental health monitoring	Not specified	Identification of relapses in people with psychotic disorders, potential for early intervention	Privacy concerns in mental health monitoring
Jahan et al. (2023) (13)	Smartwatch technology for activity recognition in religious rites	Not specified	Flexible remote monitoring beyond health and fitness, meeting cultural and religious requirements	Customization of activity detection algorithms
Bozdog et al. (2021) (14)	Remote monitoring in human behavior analysis	Wearable sensors, machine learning	Potential for understanding intricate patterns in human behavior, applications in risk prediction and coaching	Ethical and privacy concerns in human behavior analysis
Kalpana et al. (2022)	Deep learning methods for anomaly identification in human activity recognition	Not specified	Overview of current research trends, advantages, and disadvantages of various algorithms	Areas that show promise for further investigation

## 3 Proposed methodologies

The algorithmic strategies for detecting medical anomalies are:

- *Unsupervised anomaly detection*: It does not involve any supervision signal that would indicate whether a sample is normal or not during the learning process. Unsupervised methods are therefore intriguing to the machine learning field since they do not require labelled datasets. The following subsections introduce two popular unsupervised deep anomaly architectures: Autoencoders (AEs) and Generative Adversarial Networks (GANs). AEs have been extensively used for automatic feature learning ever since they were first introduced as a pre-training method for deep neural networks. The model is trained to reconstruct the input using a learnt compressed representation that is stored at the core of the architecture because the AEs are symmetrical. Assume that the current input (I) is a dataset made up of samples, and that the encoder and decoder networks are denoted, respectively. Next, the compressed form is provided as follows: Formally, let us assume that the encoder and decoder networks are denoted, that the dataset comprises samples, and that the current input is p. Next, it is decided what the compressed representation using Equation 1.

(1) $$l=h\left(i\right)$$

and the reconstruction is performed using Equation 2.

(2) $$y=g.\left(l\right)$$

To minimize the reconstruction loss, $K,pK\;\mathrm{and}\;L(x,g\left(h\left(x\right)\right)$ this model has been trained.

- *Supervised anomaly detection*: Due to its high demand in diagnostic application because of its high sensitivity and durability supervised learning is being applied widely for medical anomaly detection. It also has proven to be better performing than unsupervised methods. In This approach, a supervised signal is presented which indicates whether the samples are from the normal category or abnormal. Thus, making the job to behave as a binary classifier, and training the models using binary cross-entropy loss. Multi-task learning (MTL) which is a subtype of supervised learning, helps to transfer pertinent knowledge collected from various linked tasks, among them. For example, the difficulties brought on by subject-specific differences can be solved using a secondary subject identification task. As a result, the model develops the ability to classify anomalies while also learning to recognize similarities and differences among participants. The deep learning architectures that have been explored thus far are feedforward designs, meaning that data moves from input to output in a single direction. Their capacity to model temporal signals is hence constrained. Recurrent Neural Networks are used to overcome this restriction.
- *Recurrent neural networks (RNNs)*: Recurrence is a crucial characteristic for tasks like time-series modeling since it essentially means that the output of the current time step is once more used as an input to the subsequent time step. The modeling of sequential medical data, such as EEG and phonocardiographic data, is also essential for obtaining the temporal evolution of the signal. For modeling long-term dependencies, simple RNN architectures are ineffective due to BPTT-caused disappearing gradients. Several variations of RNN models have been created to mitigate this issue. However, because RNNs have a lot of vanishing gradients, they cannot accurately represent long-term dependencies; for this reason, LSTM networks are developed.

### 3.1 How do smart watches analyze heart rate?

**Figure 3:**
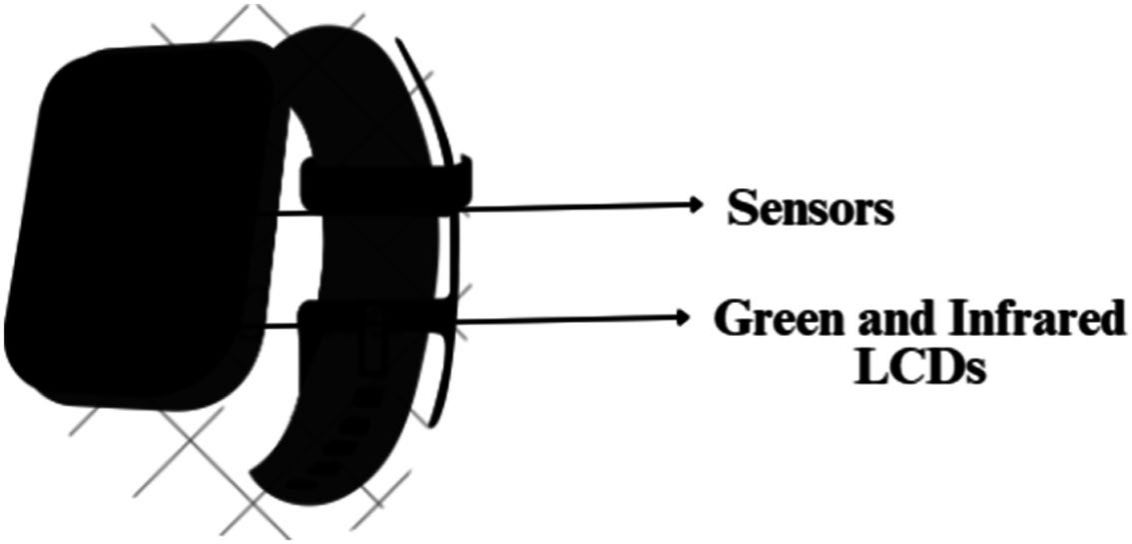
Photosensitive diodes (sensors), green LED and infrared lights on the base of a smartwatch.

The heart rate monitor of the smartwatch uses an easy and economic optical method PPG. It is employed to find changes in blood volume in the tissue’s microvascular network. This technique uses a combination of green LED and infrared light along with photosensitive diodes to illuminate the skin and measure the absorption of the green light accordingly (15) as depicted in Figure 3. Here, this combination of infrared light and green LED is taken because the Red Blood Cells (RBCs) reflect the red light and absorb the green light. This helps the sensors compute the amount of blood flowing through the wrist at any given time. The green LED is generally used when the user is performing any exercise. Normally, the watch uses infrared light to calculate the heart rate every 10 min. Furthermore, if the watch is loosely worn or the skin is perfused the LED increases its brightness and sampling rate to measure the exact heart rate. When the watch measures the heart rate every 10 min, it switches to the green LED in case the infrared light fails to provide an adequate reading (16). These lights flash hundreds of times per minute to get a hold of the blood flow which helps the device calculate the heart rate precisely.

### 3.2 Architecture of the anomaly detection model and its role in data processing of the MIMIC database

The MIMIC database (17), which contains a variety of data gathered from ICU patients, is used to calculate the relationship between PPG and ABP and assess how the model responds to abrupt variations in blood pressure. In this application, LSTM and CNN are combined. Since CNN can extract deep features and LSTM can learn from past experiences, these models are ideal for anomaly detection. Due to fully connected layers and connectionless nodes processing a single input between layers, the 2D CNN and LSTM model offers a superior classification (18). A temporal sequence is used as the input for an LSTM and is connected to the nodes from a directed graph along with a typical order.

#### 3.2.1 Convolutional neural network (CNN)

CNN is used in many different applications, such as image classification, object recognition, and medical image analysis. CNN is mainly used to extract local characteristics from higher-level inputs. These characteristics are then forwarded to lower layers for help with more complex features. Its three layers are pooling, fully connected (FC), and convolutional (FC) (1).

- Convolutional layer:

A collection of kernels for generating a tensor of feature mappings is present in the convolutional layer of the CNN layer. The kernels use the striding process to entwine the entire input to produce the output volume’s dimensions as integers, while the convolutional layer reduces the dimensions of the input volume. To retain the size of the input volume using low-level characteristics while padding an input volume of zeros, the striding procedure is required. The convolutional layer’s function is described as in Equation 3.

(3) $$G\left(x,y\right)=\left(M*N\right)\left(i,j\right)=\sum \sum M\left(x+i,y+j\right)N\left(i,j\right)$$

where G is the result of a 2D feature map, N is a 2D filter of size i × j, and M is the input matrix.

- Rectified linear unit (ReLU) layer:

The convolutional layer’s operation is indicated by M*N. Feature maps can be made more nonlinear by using the ReLU layer. ReLU uses a threshold input of zero to calculate activation. The mathematical expression for it is as follows as given in Equation 4.

(4) $$f\left(x\right)=\max\left(0,x\right)$$

- Pooling layer:

The pooling layer performs a down sample of the specified input scale to minimize the number of factors. Max pooling is the most popular technique since it yields the highest result for a certain input region. Using the characteristics gathered from the previous two layers, the FC layer computes the judgments made by CNN. It serves as a classifier, the FC layer.

- Why use ResNet?

Res Nets help in preserving a low error rate in the deeper layers of the network hence, making them one of the most efficient Neural Network Architectures. It employs a method known as skip connections (12). The salient characteristic of this technique is that regularization bypasses any layer that reduces or impedes the architecture’s performance. To form a residual block, this connection skips some layers between the activations of one layer and those of subsequent layers. These residual blocks are stacked together to create ResNets. leads to training the deep neural network without any vanishing or exploding gradient disruptions. This network fits the residual mapping by letting the network do the fitting. Hence, instead of saying G(x), initial mapping, let the network fit as defined by Equation 5.

(5) $$H\left(i\right):=G\left(i\right)-H\left(i\right):=G\left(x\right)+i$$

#### 3.2.2 Long short – term memory (LSTM)

**Figure 4:**
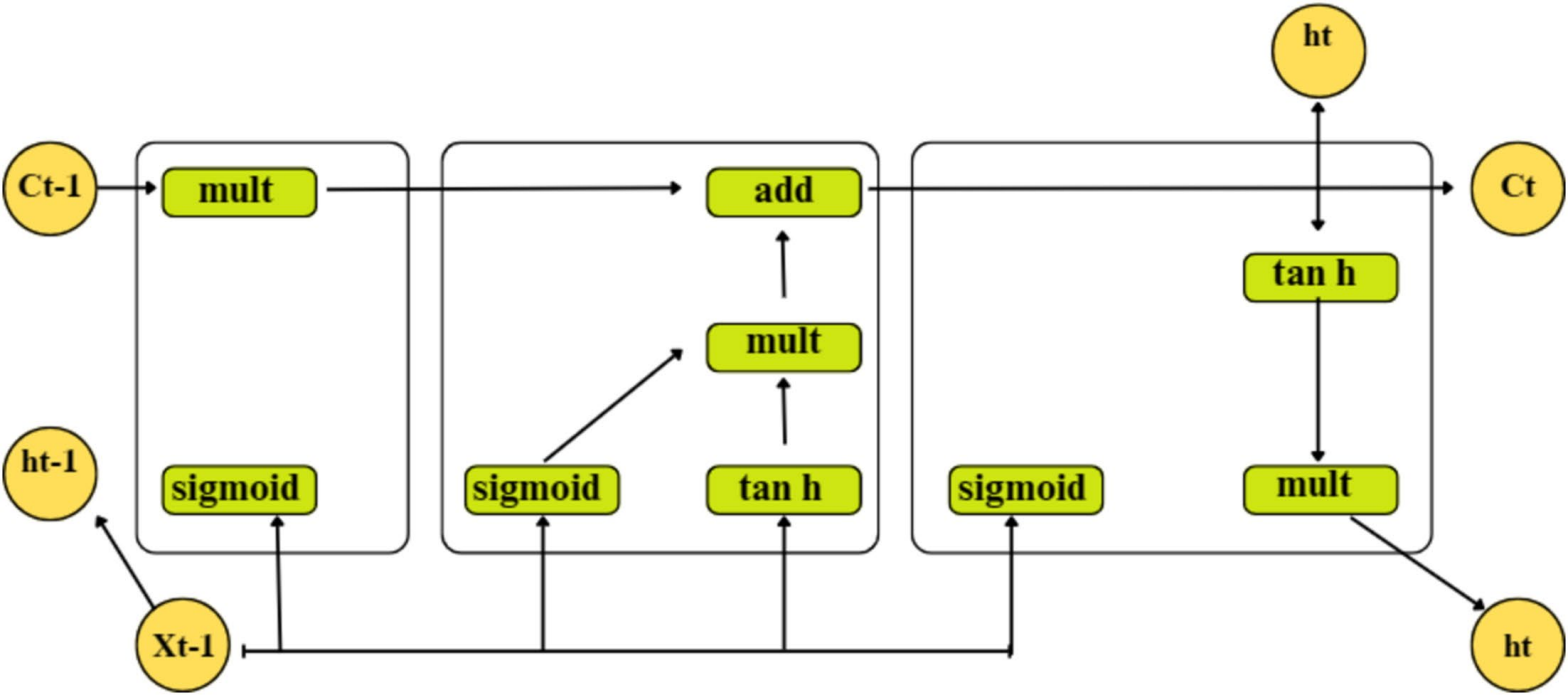
Architecture of LSTM input gate.

A particular kind of recurrent neural network called an LSTM solves disappearing and exploding gradient problems by using memory blocks rather than the standard RNN units. The LSTM’s cell state also stores the long-term states, enabling it to link data gathered in the past and present. Three distinct types of gates make up the internal structure of the LSTM, as shown in Figure 4:

$x_{p}$ - denotes the current input;

$C_{p}$ and $C_{p-1}$ – denote the new and previous cell states, respectively; and

$h_{p}$ and $h_{p-1}$ - denote the current and previous outputs, respectively.

(6) $$i_{p}=\sigma \left(W_{i}.\left[h_{p-1},x_{p}\right]+b_{i}\right)$$

(7) $${\tilde{C}}_{p}=\tanh\left(W_{i}.\left[h_{p-1},x_{p}\right]+b_{i}\right)$$

(8) $$C_{p}=f_{p}\;C_{p-1}+i_{p}{\tilde{C}}_{p}$$

(9) $$f_{p}=\sigma \left(W_{f}.\left[h_{p-1},x_{p}\right]+b_{f}\right)$$

Where, ${\tilde{C}}_{p}$ represents the current moment information and refers to a tan h output,

$C_{p-1}$ represents the long-term memory information,

$W_{i}$ denotes a sigmoid output and the weighted matrices of the input gate,

$b_{i}$ represents the LSTM bias of the input gate,

$W_{f}$ represents the weight matrix,

$b_{f}$ represents the offset, and

σ represents the sigmoid function.

Here, $x_{p}$ and $h_{p-1}$ are passed through a sigmoid layer using Equation 6. to determine which portion of the data needs to be added. Moreover, after $x_{p}$ and $h_{p-1}$ have passed through the tanh layer, Equation 7 is utilized to extract new information, and Equation 8 integrates long-term memory and current memory into $C_{p}$ . The information from a previous cell that can be forgotten is determined using Equation 9.

(10) $$O_{p}=\sigma \left(W_{o}.\left[h_{p-1},x_{p}\right]+b_{o}\right)$$

(11) $$h_{p}=O_{p}\tanh\left(C_{p}\right)$$

Where, $W_{o}$ represents the weighted matrices of the output gate, and.

$b_{o}$ represents the LSTM bias of the output gate.

Using Equations 10, 11, the output gate determines the states necessary for $x_{p}$ and $h_{p-1}$ inputs to continue. To obtain the final output, the state decision vectors that transfer new information, Ct, across the tanh layer are located and multiplied.

#### 3.2.3 Combined CNN-LSTM network

**Figure 5:**
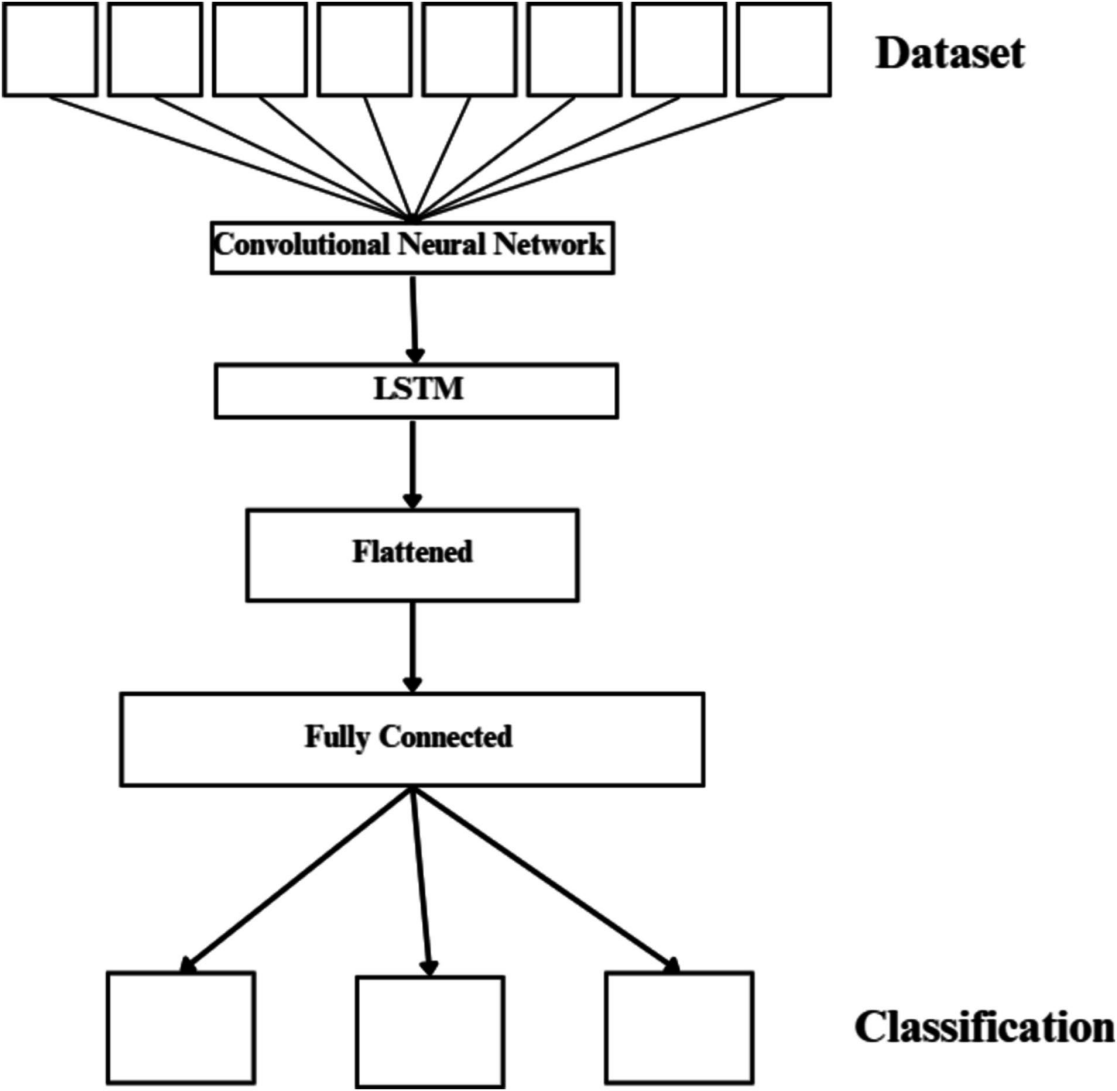
Proposed hybrid network.

This design uses the LSTM as a classifier and the CNN to extract complex features from images (19). The suggested network has a total of 20 layers, as seen in Figure 5. These layers consist of an FC layer, an LSTM layer, five pooling layers, twelve convolutional layers, and an output layer that applies the SoftMax function. Two or three 2D CNNs, a pooling layer, and a dropout layer with a 25% dropout rate connect each convolutional block. The 3×3 sized kernel convolutional layer is activated by the ReLU function and prepared for feature extraction. The max-pooling layer’s 2×2 size kernels are used to reduce the size of the input image. The LSTM layer uses the function map transferred in the last stage of the model to extract time information.

### 3.3 How the hybrid network identifies high-risk ABP conditions

There are two approaches to detect hypertension and monitor the blood pressure. The first method treats the model as though it were a regression task—that is, as though it produces continuous values. As a result, the systolic and diastolic values of blood pressure are calculated using the PPG and ECG signals (20). By using both signals or features computed from PPG and ECG (or, in some cases, only PPG signal is used) signals as input, various machine learning techniques, such as linear regression models and artificial neural networks (ANN) for regression tasks, are used to estimate BP values.

The second approach treats the model as if it generates discrete values or labels, i.e., like a classification task. In this method, the models try to compute the level of hypertension the patient belongs to, based on clinical and socio-demographic data (21). This approach differs from the first is that, the first approach utilizes raw signals or features extracted from the input data used in ML model, whereas the second approach makes use of continuous clinical data.

- Regression task (first approach)

Linear regression formula is defined in Equations 12, 13:

(12) y=mx+b

Where:

- *y* is the predicted blood pressure value.
- *m* is the slope.
- *x* is the input signal.
- *b* is the y-intercept.

- Classification task (second approach)

SoftMax function:

(13) $$\mathrm{SoftMax}{\left(x\right)}_{i}=\frac{e^{\mathrm{xi}}}{\sum_{j}e^{xj}}$$

where:

- *e* is the base of the natural logarithm.
- *x*_i_ is the input value for class *i*.
- The function outputs a probability distribution over multiple class.

The following subsection provides insights on how to use these methods along with transfer learning in the hybrid network.

### 3.4 How to integrate transfer learning in the hybrid network

**Figure 6:**
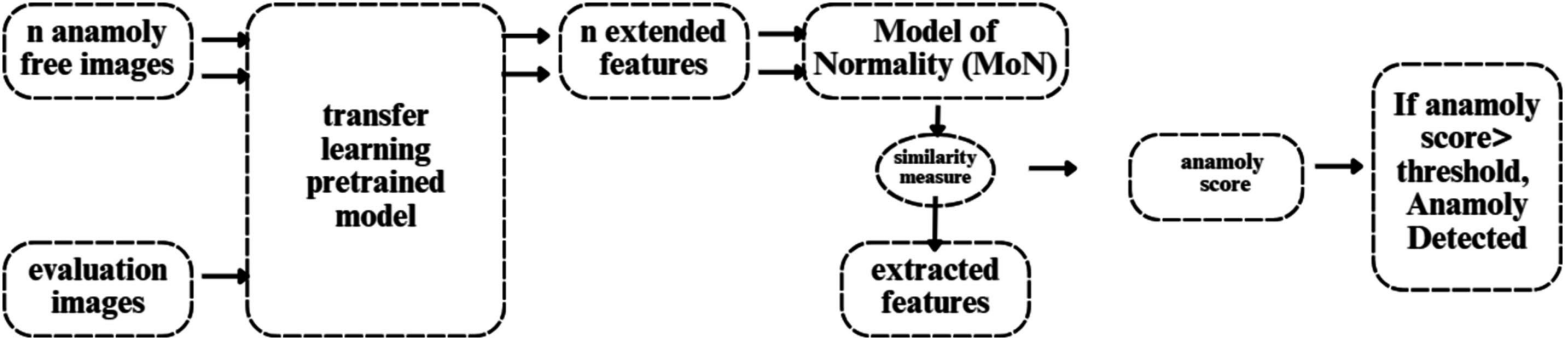
Anomaly detection.

Figure 6 illustrates the flowchart for anomaly detection, which feeds N anomaly-free images into the deep feature extractor of the transfer learning model. The MoN, which learns/extracts normality from the input images, is created using the learnt or extracted features. Consequently, a transfer learning model is used to extract the features for a given input image. A similarity measure is then used to compare the extracted features to the MoN, and the anomaly is identified if the resulting anomaly score is greater than the decision threshold.

- Transfer learning model

We use EfficientNet, which was trained on the ImageNet dataset, for transfer learning. It employs a state-of-the-art scaling method that uniformly scales each dimension (depth, width, and resolution) using a compound scaling coefficient. The balanced scaling of the model leads to improved performance. The baseline network of EfficientNet, called “EfficientNet-B0,” maximizes FLOPS and precision (22). Next, the baseline network was scaled with different compound coefficients to create the “EfficientNet-B1 through B7” EfficientNet scaled versions. Using a multi-objective Neural Architecture Search (NAS) that improves accuracy and FLOPS, the core network of EfficientNet was built.

- Model of normality (MoN)

The representations that do not fit its specification are marked as anomalies since MoN learns normality from the characteristics that are extracted. Therefore, all regular variants must be included in the MoN creation for the designated purposes. A MoN is constructed for each data class by averaging the learnt features taken from the N normal pictures, which are only used to create MoNs and are not included in the evaluation set.

- Similarity measure

The MoN’s subjective similarity to an image can be expressed in terms of a distance measure specified on the learnt feature space, since each input image’s deep-learned features function as a unique identifier. In order to accomplish this, we use Euclidean distance to calculate the similarity between MoN and features taken from the test photos.

Euclidean distance formula is given in Equation 14:

(14) $$\mathrm{Distance}\;\left(\mathrm{MoN,Image}\right)=\sqrt{\underset{i}{\sum }{\left(MoN_{i}-\mathrm{Imag}e_{i}\right)}^{2}}$$

**Figure 7:**
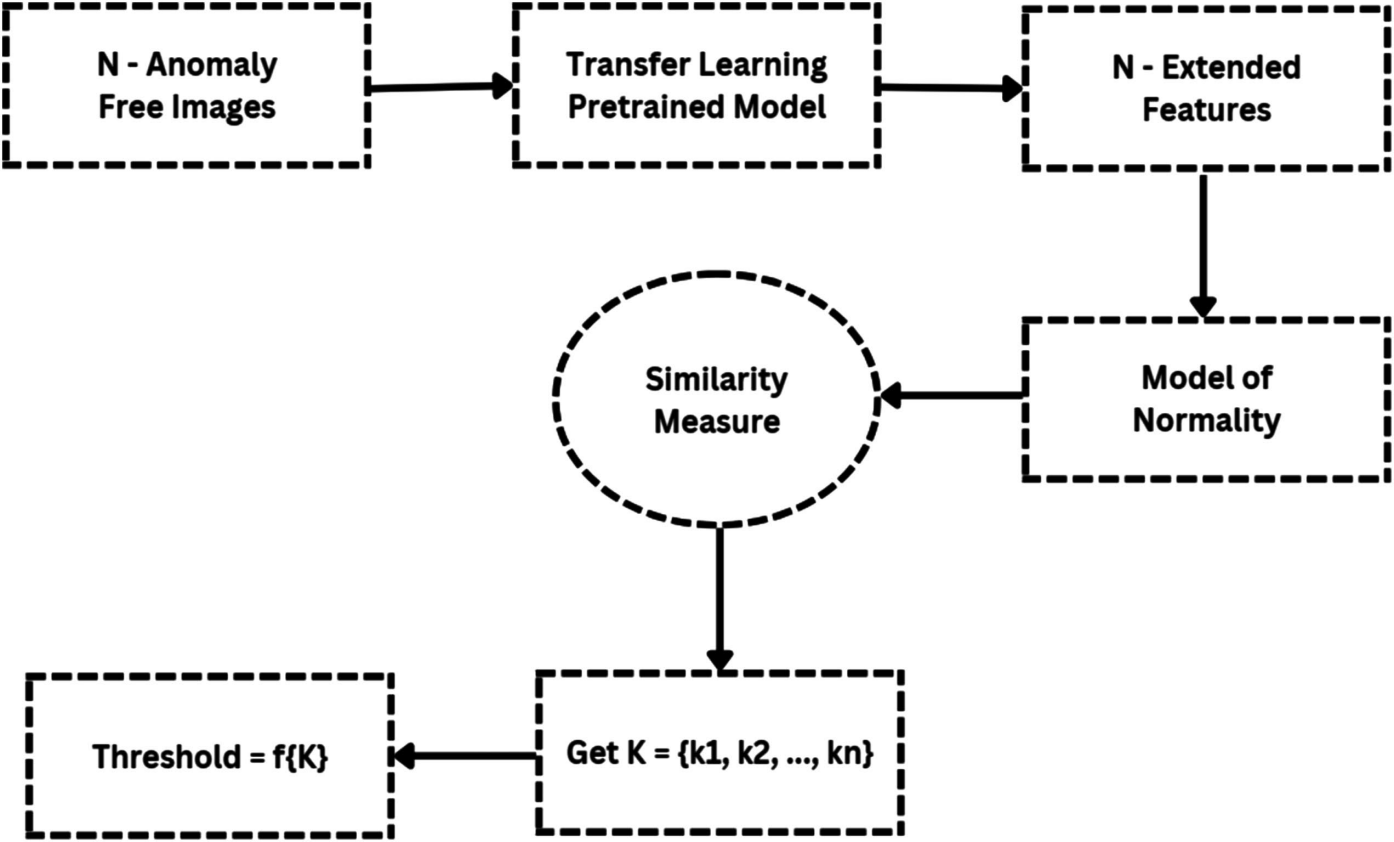
Working-point threshold setting process.

Since it directly impacts detection efficiency, the decision threshold is a critical component of distance-based anomaly detection algorithms. Figure 7 shows a flowchart that illustrates the threshold-setting procedure. By adjusting this level appropriately, it is possible to significantly increase detection accuracy while reducing false positive rate. Here, based on the vectors K_max_ and K_mean_, we suggest a clear way for setting the working-point threshold in Table 4.

**Table 4:** Working-point threshold based on the vectors K_max_ and K_mean_.

Threshold (T)	K_1_ - K_max_, K_2_ – K_mean_
T_1_	max(K_1_)
T_2_	max(K_1_) - std.(K_1_)
T_3_	mean(K_1_) + std.(K_1_)
T_4_	max(K_2_)
T_5_	max(K_2_) - std.(K_2_)
T_6_	mean(K_2_) + std.(K_2_)

### 3.5 About dataset

**Figure 8:**
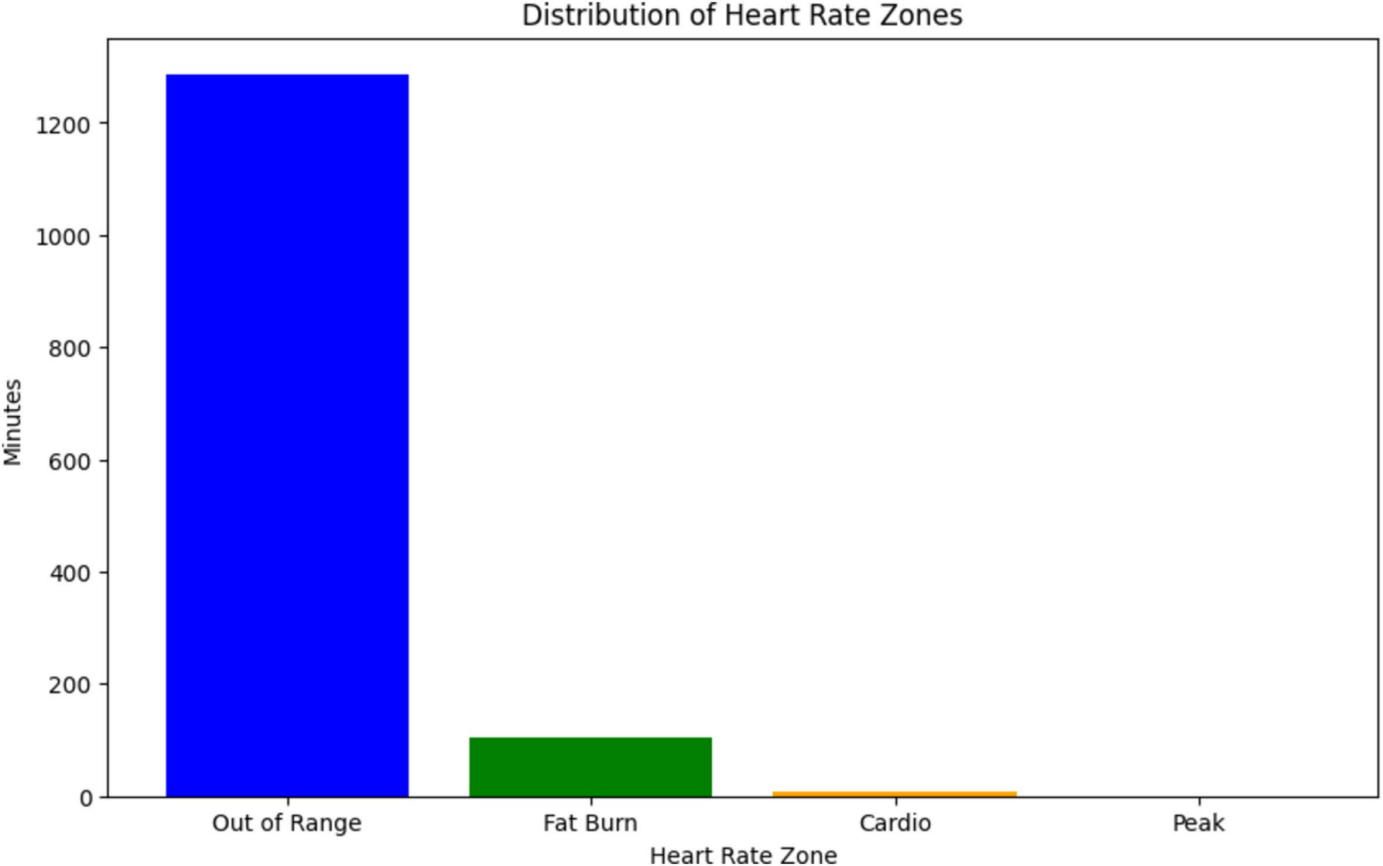
Distribution of heart rate zones as per our readings.

The graph illustrated in Figure 8, shows the distribution of heart rate zones recorded during a specific date (2019-04-11) as part of SHM using wearable sensors, from the dataset (23). Each heart rate zone, categorized based on intensity levels such as “Out of Range,” “Fat Burn,” “Cardio,” and “Peak,” is represented by a bar in the graph. The height of each bar corresponds to the duration (in minutes) spent in the respective heart rate zone, while the color coding helps distinguish between different zones.

This graph is particularly relevant to our discussion on the use of physiological signals, such as ECG and PPG, in predicting arterial BP through neural network architectures (24, 25). In the context of ResNet-LSTM network’s superior performance in health monitoring, this graph provides valuable insights into the distribution of heart rate zones, which are indicative of the intensity levels of physical activity or exertion (26–28). Understanding these heart rate patterns can contribute to the accurate prediction of BP and overall health monitoring (29). Furthermore, the graph aligns with the significance of anomaly detection and the need for accurate monitoring through wearables. By analyzing heart rate data and identifying anomalies or irregularities in heart rate patterns, healthcare professionals can intervene in a timely manner to address potential health concerns (30, 31). Overall, this graph serves as a visual representation of the physiological data collected through SHM, supporting the discussion on leveraging innovative technologies for real-time health monitoring and prediction (32–34).

The MIMIC (Medical Information Mart for Intensive Care) dataset is a critical resource in this research, providing a diverse collection of real-world clinical signals that support the development and evaluation of the proposed blood pressure (BP) prediction models. MIMIC contains rich physiological data, including Electrocardiogram (ECG), Photoplethysmography (PPG), and Arterial Blood Pressure (ABP) measurements from a wide variety of patients with different medical conditions (35, 36). This diversity allows models like the ResNet-LSTM to generalize across various patient profiles and medical scenarios, improving the accuracy and reliability of BP anomaly detection. In the study, the dataset was used to train and validate machine learning models designed to predict systolic and diastolic BP. The results, displayed in Tables 5, 6, show that the ResNet-LSTM model outperforms other architectures in terms of Mean Absolute Error (MAE) and Root Mean Squared Error (RMSE). This comprehensive dataset played a crucial role in the testing and validation of the proposed model, ensuring that the models were exposed to real-world complexities, thus enhancing their predictive power and applicability in clinical settings.

**Table 5:** Errors on the total BP prediction using the MIMIC database.

Tested set	MAE	RMSE	MAE	RMSE
Dataset	Photoplethysmography dataset		Photoplethysmography + Electroencephalogram Dataset	
Fully connected	18.6	27.2	18.3	25.7
Long Short-Term Memory	8.6	13.3	5.9	9.3
WaveNet	12.3	18.3	11.3	17.5
WaveNet + Long Short-Term Memory	10.0	15.6	5.7	9.0
ResNet + Long Short-Term Memory	6.2	8.9	3.3	5.0

**Table 6:** LOO outcomes using ResNet-LSTM on the MIMIC database.

Tested set	RMSE	MAE	MAE D	MAE S	RMSE D	RMSE S
Direct SBP/DBP prediction						
Photoplethysmography (50 pat)	-	-	10.746	23.598	12.344	27.643
Photoplethysmography (40 pat)	-	-	11.106	24.223	12.642	28.247
Electrocardiogram (40 pat)	-	-	9.548	20.367	10.848	23.070
Entire BP prediction						
Photoplethysmography (50 pat)	19.155	15.342	10.684	21.467	12.349	25.383
Photoplethysmography (40 pat)	19.560	15.679	10.818	22.409	12.411	26.246
Electrocardiogram (40 pat)	18.018	14.609	10.105	22.099	11.529	24.587

## 4 Results

The results of BP prediction achieved using the distinct settings and networks are summarized in Table 5. Performance was improved in each setup when PPG was used. The ResNet + LSTM network, which accurately predicted BP values, was the best one (Table 6). On the validation set, the network overall MAEs were considered. Since the networks are designed with the primary objective of generating BP values in mind, direct BP prediction appears to be the optimal strategy. But when networks need to infer the entire signal, they need to learn information that will not be used. Table 7 illustrates that the ResNet + LSTM is the optimal network in both scenarios and also illustrates the neural network’s complexity for anomaly detection.

**Table 7:** Comparison of complexity of various neural networks.

Neural network	Cost estimation (FLOPs)		Complexity order
	PPG + ECG	PPG	
Fully connected	∼2,500	∼625	O(L × (V_dim_)^2^)
Long Short-Term Memory	∼2,500	∼625	O(L × (V_dim_)^2^)
WaveNet	∼7,500	∼1850	O(L × (V_dim_)^2^ × k_size_)
WaveNet + Long Short-Term Memory	∼7,500	∼1850	O(L × (V_dim_)^2^ × k_size_)
ResNet + Long Short-Term Memory	∼17,500	∼4,375	O(L × (V_dim_)^2^ × k_size_)

The errors in the total BP prediction for various setups using the MIMIC database (17) are displayed, as shown in Supplementary Figure S5. MAE and RMSE values are shown in the figure to illustrate how different configurations—Fully connected, LSTM, WaveNet, WaveNet+LSTM, and ResNet+LSTM—perform in terms of performance. As stated in the research of Paviglianiti et al. (5) titled “A Comparison of Deep Learning Techniques for Arterial Blood Pressure Prediction” published in Cognitive Computation, statistical comparisons with current models were conducted for more thorough examination. Using a custom dataset from the works of Paviglianiti et al. (5) and ECG, PPG, and ABP readings taken from the MIMIC database, our model is trained and tested. This collection of clinical signal data is a priceless resource that provides an accurate representation of physiological parameters observed in daily life. With the use of this vast and varied dataset, our models were able to learn and generalize across a broad range of medical conditions and patient profiles. We ensured that our models were exposed to the nuances and complexities found in actual patient data by utilizing clinical signals from the MIMIC database, which increased their predictive power.

The MIMIC Database’s Leave-One-Out (LOO) results are shown in Supplementary Figure S6, with an emphasis on the optimal neural network architecture, ResNet-LSTM. The figure shows whole BP prediction scenarios as well as MAE and RMSE values for direct systolic/diastolic blood pressure (SBP/DBP) prediction using PPG and ECG signals.

In addition, we validated our models’ performance using the Pulse Transit Time PPG dataset. To ascertain whether our models could be used outside of the training set, this additional dataset was essential. Carefully comparing the results to this independent dataset demonstrated the robustness and reliability of our models. Consequently, we were able to assess our models’ efficacy against state-of-the-art techniques and gain a better understanding of how well they predicted arterial blood pressure.

**Figure 9:**
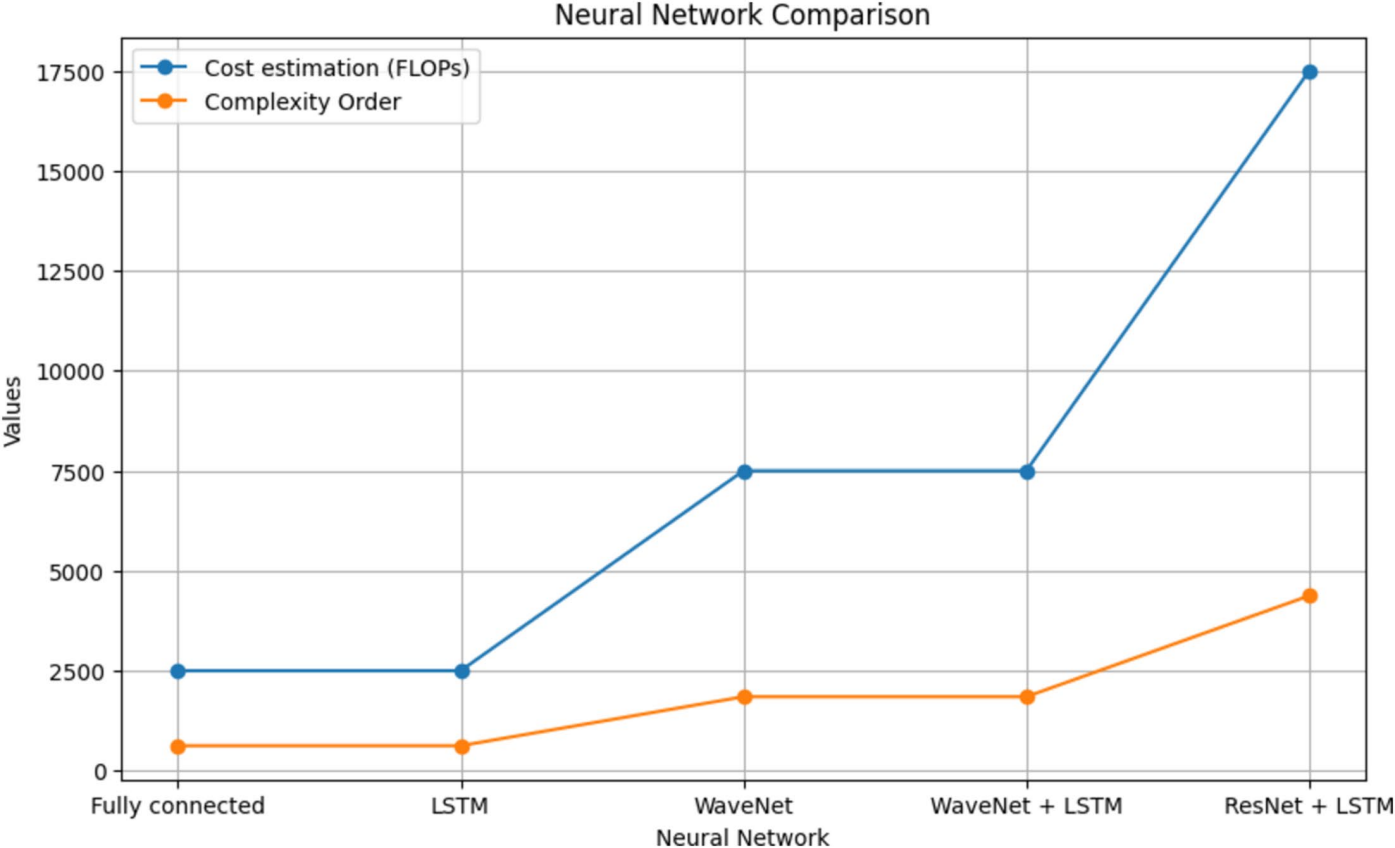
Neural network comparison.

A thorough comparison of neural network complexities is shown in Figure 9. The graph shows the cost estimation and complexity order (FLOPs) for several neural network architectures, such as ResNet+LSTM, WaveNet, WaveNet+LSTM, and Fully connected. Understanding each architecture’s computational efficiency is made easier with the help of this visual representation.

**Figure 10:**
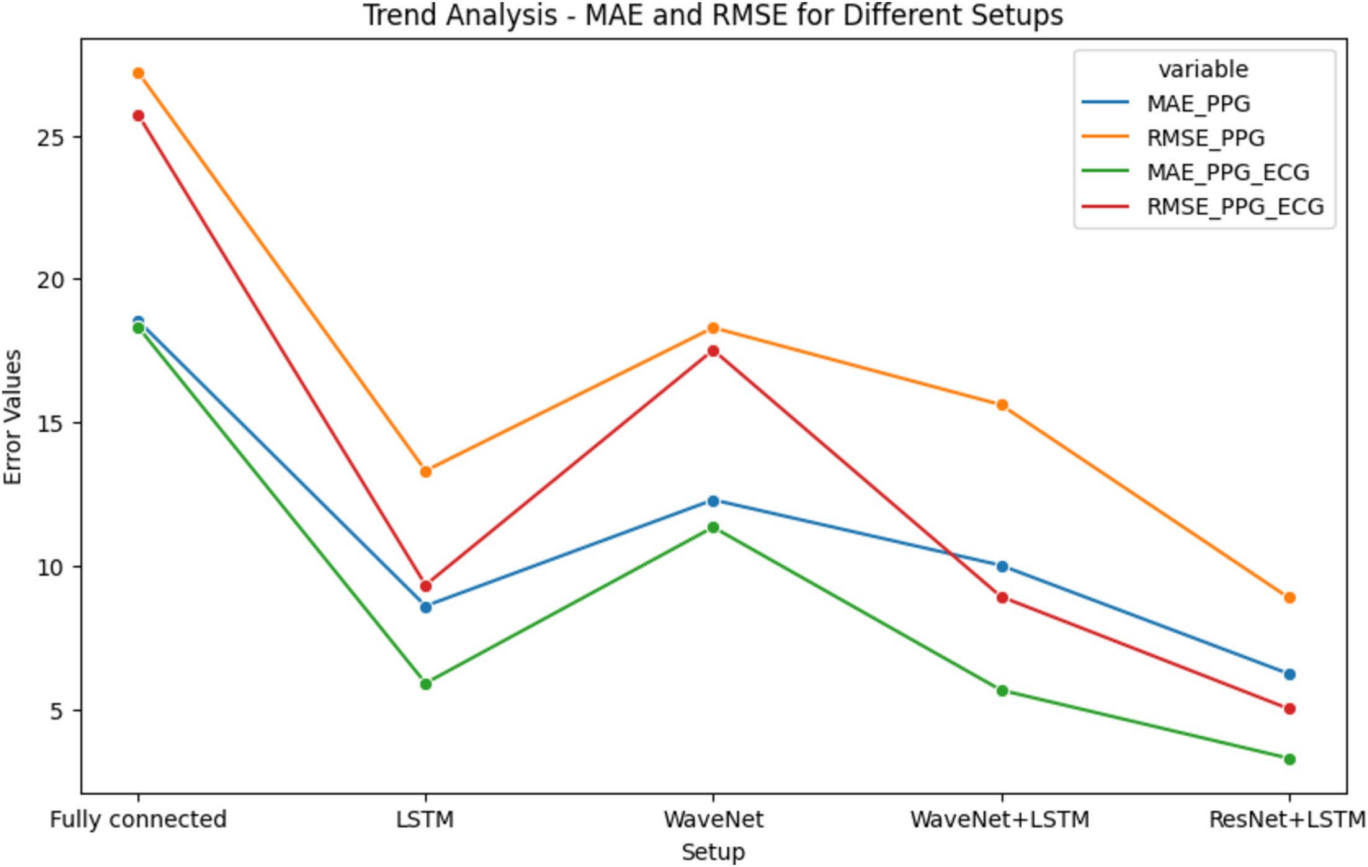
Trend analysis of MAE and RMSE for different setups.

Figure 10’s-line plot illustrates the trend of MAE and RMSE in several configurations, such as Fully Connected, LSTM, WaveNet, WaveNet+LSTM, and ResNet+LSTM. The x-axis represents the different setups, and the y-axis shows the error values. Plot performance for each setup in terms of error distribution and prediction accuracy is shown. The MAE and RMSE for direct Systolic Blood Pressure and Diastolic Blood Pressure predictions are compared in the bar chart shown in Figure 10 for several tested sets, such as PPG (50 pat), PPG (40 pat), and ECG (40 pat). The graphic aids in evaluating how well the neural network (ResNet+LSTM) predicts blood pressure based solely on physiological signals.

**Figure 11:**
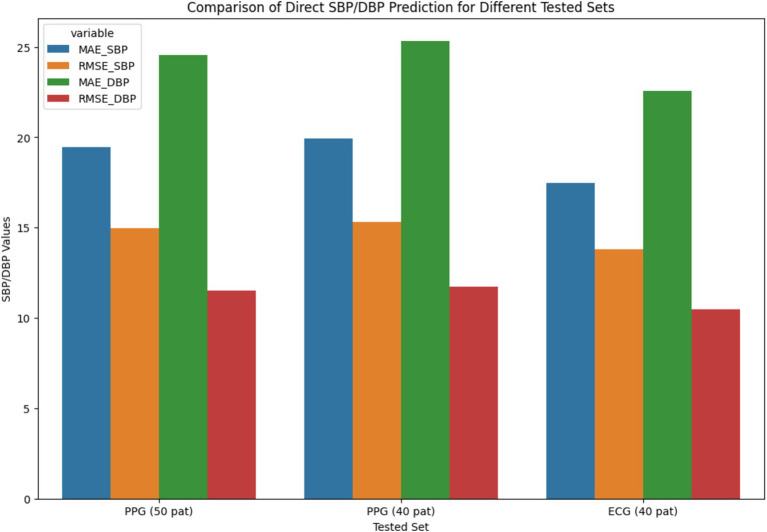
Comparison of direct SBP/DBP prediction for different tested sets.

**Figure 12:**
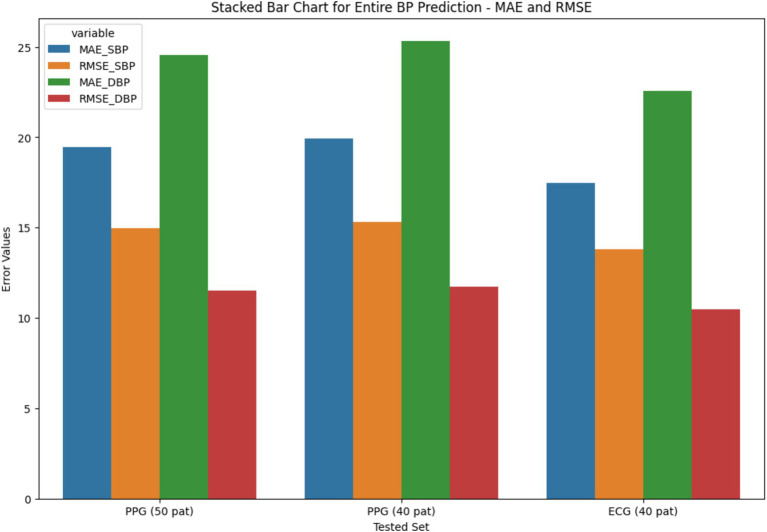
Stacked bar chart for entire BP prediction based on MAE and RMSE.

The stacked bar chart as depicted in Figures 11, 12, illustrates the MAE and RMSE for entire blood pressure prediction across different tested sets. The chart is divided into segments representing MAE and RMSE values for direct SBP and DBP predictions using the ResNet+LSTM neural network. It offers a visual comparison of the errors associated with different physiological signals and tested sets.

**Figure 13:**
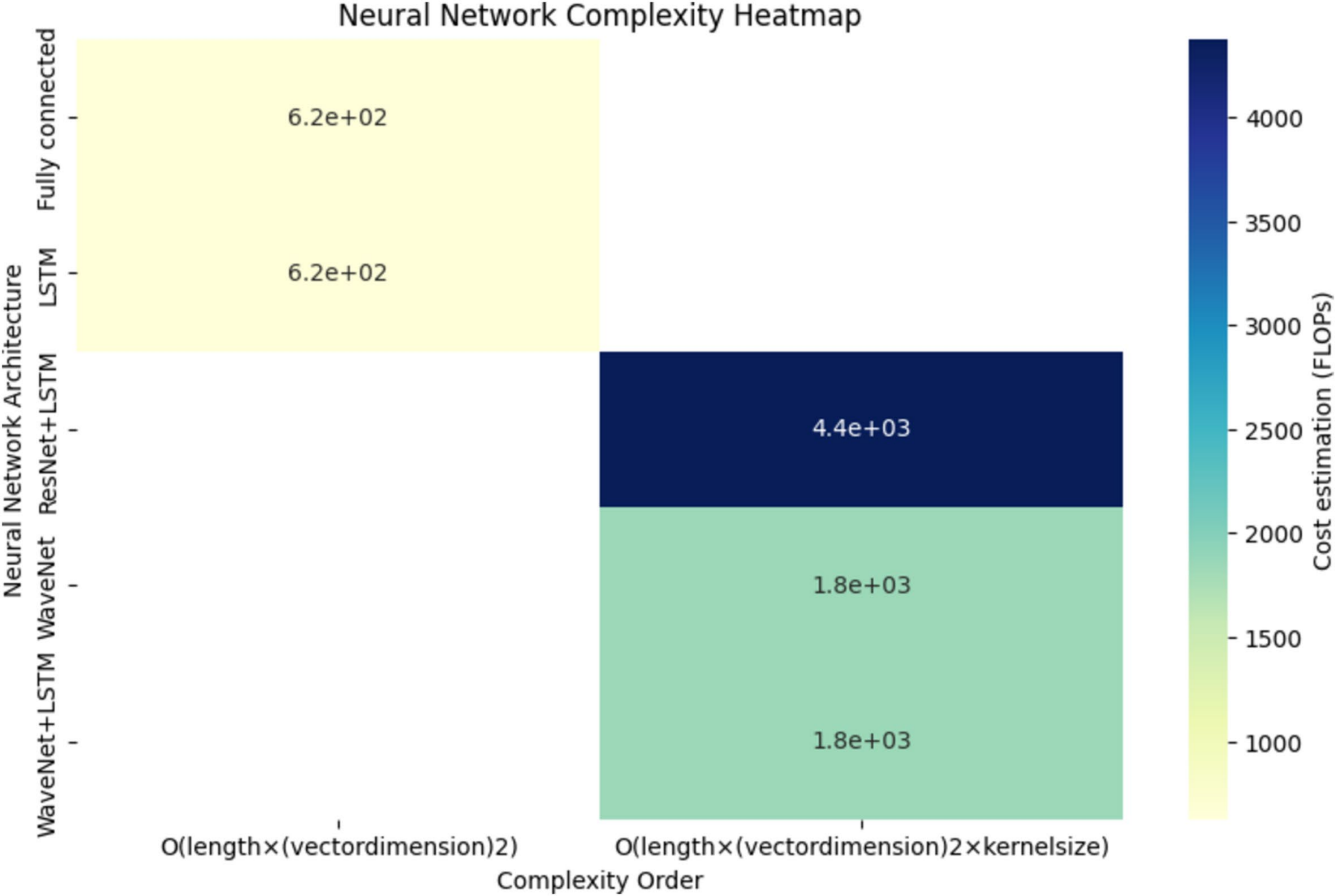
Neural network complexity heatmap.

The heatmap depicted in Figure 13, provides a comprehensive overview of the computational complexity (Cost estimation in FLOPs) associated with different neural network architectures. Each cell in the heatmap corresponds to a specific neural network’s complexity for predicting arterial blood pressure using PPG signals. Darker shades represent higher computational costs.

### 4.1 Results from our readings

**Figure 14:**
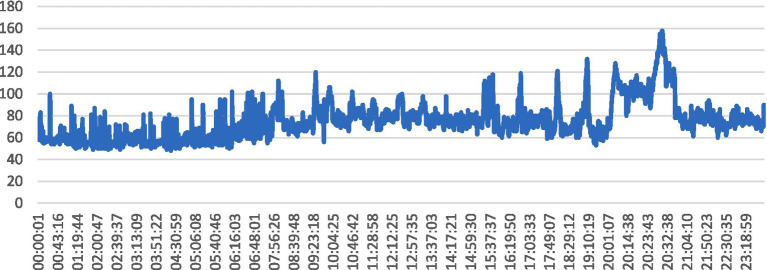
Timestamp vs. heart rate line graph from our readings.

The line graph illustrated in Figure 14, depicts the variation in heart rate values over time, captured at regular intervals during a specific monitoring session. Each data point on the graph represents a recorded heart rate value at a particular timestamp, with the x-axis indicating time and the y-axis representing heart rate values.

The graph in Figure 14 is pertinent to the abstract’s exploration of SHM and the utilization of wearable sensors for real-time health monitoring. It reflects the continuous monitoring of physiological parameters, such as heart rate, which is crucial for assessing overall health status and detecting anomalies or irregularities. It provides insights into the temporal dynamics of heart rate, which is a key physiological signal used in BP prediction models. By analyzing trends and fluctuations in heart rate values over time, healthcare professionals can infer patterns of physical activity, stress, or other factors that may impact BP levels. Changes in heart rate patterns, as depicted by the line graph, can serve as indicators of potential health concerns or deviations from normal physiological states, prompting timely interventions or further investigation.

**Figure 15:**
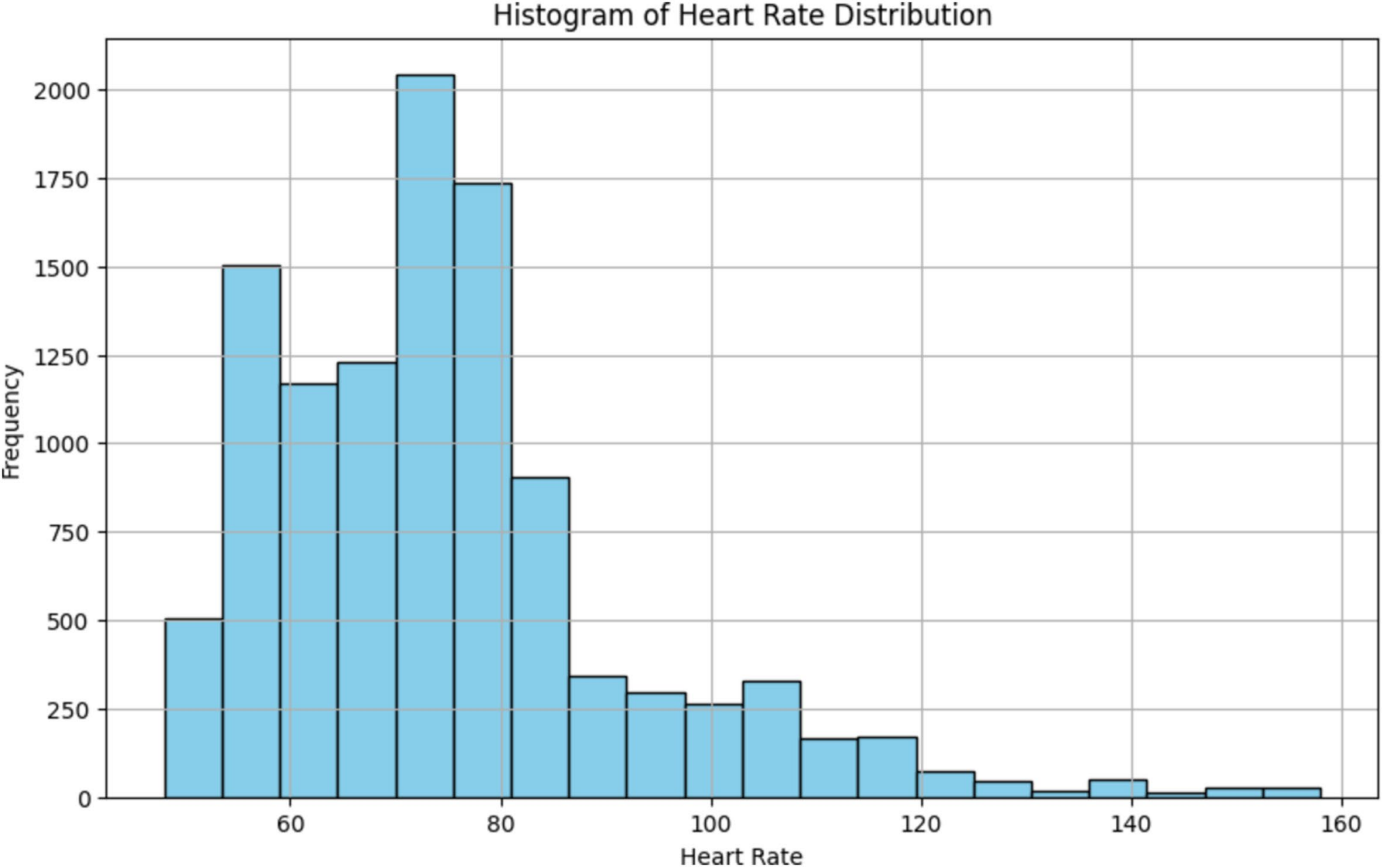
Histogram of heart rate distribution from our readings.

The histogram depicted in Figure 15, provides a visual representation of the distribution of heart rate values, allowing us to identify the central tendency and spread of heart rate data. By observing the shape of the histogram and the location of its central peak, we can gain insights into the typical range of heart rate values recorded during the monitoring session. Additionally, the spread of the histogram can indicate the variability or dispersion of heart rate values around the central tendency. Overall, the histogram provides a summary of the overall heart rate distribution, aiding in the interpretation of physiological responses and patterns observed during the monitoring session.

**Figure 16:**
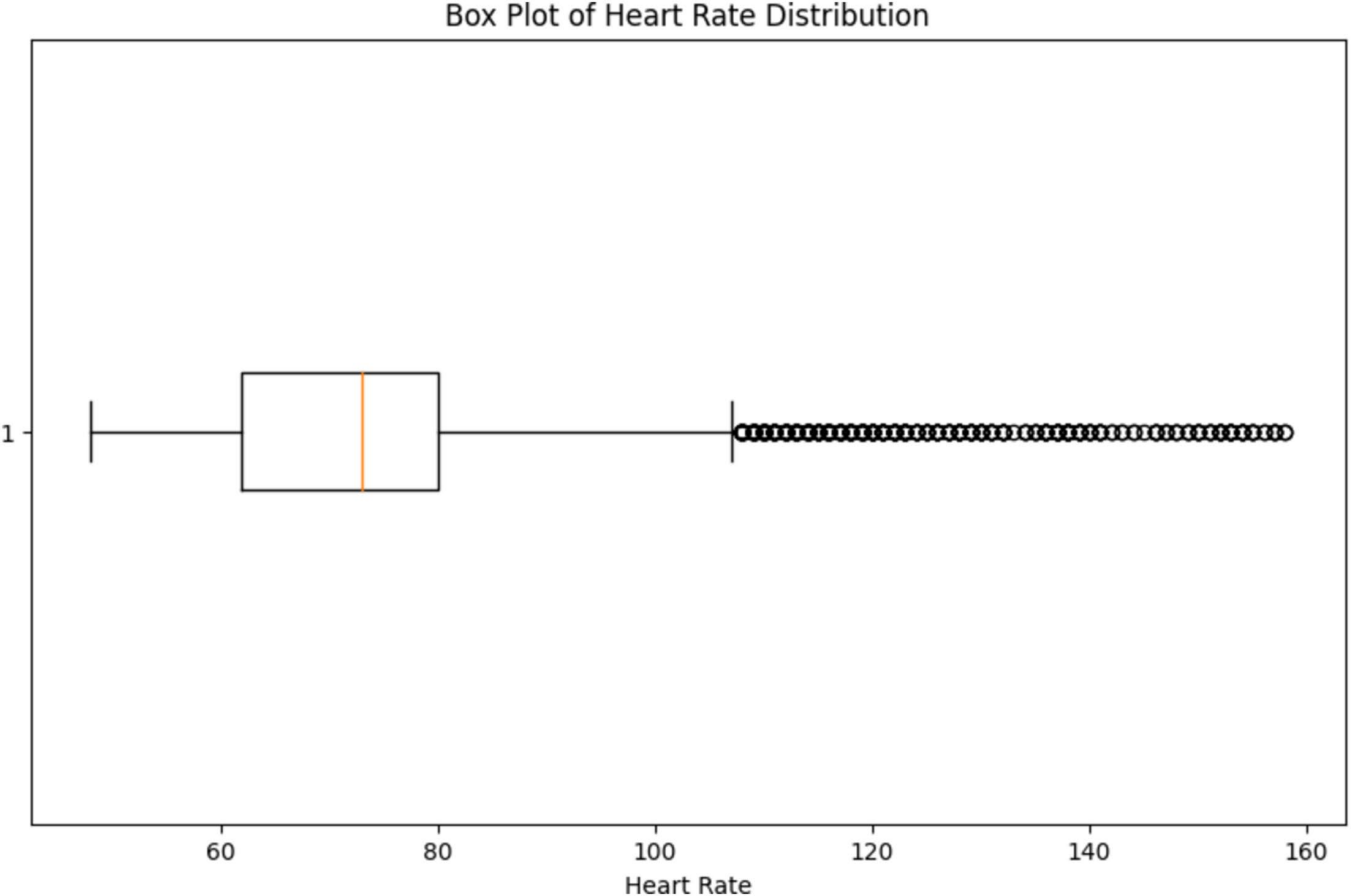
Box plot of heart rate distribution from our readings.

The box plot depicted in Figure 16, provides a visual representation of the variability in heart rate values, allowing us to assess the spread and dispersion of the data. By observing the box plot, we can identify the median (central tendency) of the heart rate values, as well as the interquartile range (IQR) which represents the spread of the middle 50% of the data. Additionally, any outliers or extreme values beyond the whiskers of the box plot can indicate potential anomalies or irregularities in the heart rate data. By visually inspecting the box plot, healthcare professionals can identify any outliers or extreme values that may require further investigation or intervention, supporting the overarching goal of continuous monitoring and early detection of health issues.

**Figure 17:**
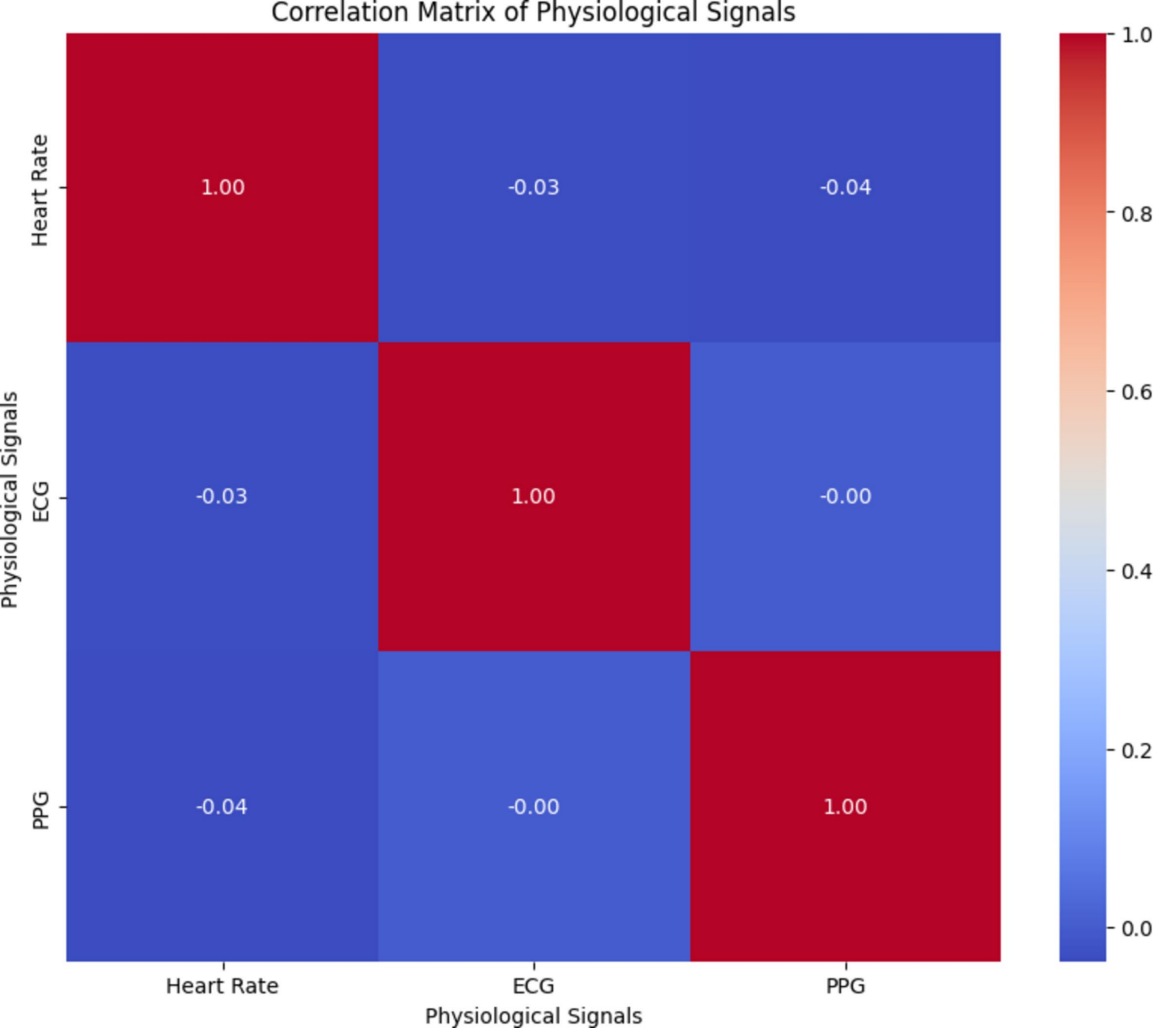
Correlation matrix of physiological signals from our readings.

By analyzing the correlation matrix illustrated in Figure 17, healthcare professionals can identify any correlations or dependencies between different physiological signals, which are crucial for accurately predicting arterial blood pressure and overall health monitoring. Understanding the relationships between physiological signals can aid in the development of effective prediction models and personalized healthcare interventions.

## 5 Conclusion

An approach to evaluate personal healthcare is to continuously monitor physiological markers. Understanding the underlying causes of illness states can be greatly aided by identifying patterns that can be discovered by recognizing outliers or irregularities in heart rates and other characteristics. The vast amount of data collected by wearable device sensors contains irregularities, hence finding anomalies requires accurately automated algorithms. Across the globe, there is a gradual but continuous transition from hospital-based care to patient-centric care. This will gradually pave the way for a data influx, along with the rise in popularity of wearable technology. Any illness status tracking requires continuous wearables-related data points, which are outside the purview of medical care. Such daily longitudinal data collection over extended times can lead to data buildup. The methods used to obtain and disseminate the data determine how the data will be used analytically, as has been described throughout this current article. It is crucial to build wearables-related software for precise health monitoring as well as cutting-edge data collection, analysis, and visualization. Exact clarifications that can be linked to activity of the user and everyday involvement are necessary for both solo and hybrid systems for anomaly identification. The transparency of algorithms used to calculate step or sleep data is another crucial area that should be supported. Enhancement of anomaly detection algorithms take place continuously. To increase the predictability huge datasets have also been made accessible simultaneously. Thus, there is a connection between wearable technology and data analysis and important sectors like the cloud and data security. A wearable device’s ability to communicate with hand-held devices like smartphones and the cloud is facilitated by the internet connection, particularly Wi-Fi.

## 6 Discussion

Numerous detection techniques have been suggested to identify anomalies due to their clinical importance and the effects they have on diagnosis and treatment. Wearable gadget clinical investigations are also becoming more common. Several essential conditions must be met to get therapeutically useful outputs from wearables-related data. To generate suggestions, users must make decisions and align their goals, which both could call for platforms in addition to mobile apps. It is important to test forth current recommendations regarding the correlation of different device-derived data. For instance, when exercise and sleep are connected, the underlying physiological imbalance can be hidden.

By building a dedicated cloud infrastructure for data analysis and storage, wearables can be used more effectively in healthcare. The security of these devices should also be considered. Device-to-device connectivity and an online cloud infrastructure subject to strict regulations are prerequisites for the digital healthcare framework. Eventually, these components might help with the use of wearable big data and accurate anomaly detection in pathology research. Apart from these factors, the next improvements ought to concentrate on the cutting-edge cloud infrastructure designed specifically for the analysis and archiving of health data generated by wearables. To ensure scalability, security, and smooth integration with healthcare systems, this infrastructure should effectively manage the substantial amount of data generated by wearables. Moreover, the creation of a networked wearable ecosystem may open new opportunities for health monitoring synergies, enabling the cooperative use of fitness trackers, smartwatches, and medical sensors to provide a thorough understanding of a person’s wellbeing.

Work on improving anomaly detection models should consider contextual integration of different wearable data streams. Comprehending the interplay among variables like ambient circumstances, user behavior, and physiological metrics can offer a more comprehensive perspective on a person’s health state. This strategy is in line with the development of user-centric decision support systems, which customize insights and suggestions based on data from wearables to personal objectives and health goals. To create a seamless data flow between wearables and healthcare providers, collaboration with current health platforms and electronic health records (EHRs) is essential. Ensuring compliance with interoperability standards promotes timely interventions based on anomaly detection results and comprehensive patient care. In addition, the incorporation of behavioral analytics into models for anomaly detection can provide a more profound comprehension of patterns concerning user behavior, way of life, and compliance with health advice. This can improve the accuracy of anomaly detection by accounting for individual behavioral differences.

Experiential validation studies should be carried out as wearable technology in healthcare continues to advance to evaluate the practicality and user-friendliness of anomaly detection systems. Algorithm modifications based on user and healthcare professional feedback can enhance the technology’s overall usefulness. The responsible and efficient integration of wearable technology in healthcare will be further aided by the creation of ethical frameworks, the adoption of explainable AI techniques, and the development of strategies for long-term health monitoring using wearables.

## Data Availability

The datasets presented in this study can be found in online repositories. The names of the repository/repositories and accession number(s) can be found at: our dataset: https://github.com/VsinK14/SHM, MIMIC dataset: https://archive.physionet.org/physiobank/database/mimicdb/.
