# Supplementary figures and images for: Detecting anomalies in smart wearables for hypertension: a deep learning mechanism
Source: Front Public Health. 2025 Jan 15;12:1426168. doi: 10.3389/fpubh.2024.1426168 (PMC11755415; doi:10.3389/fpubh.2024.1426168)

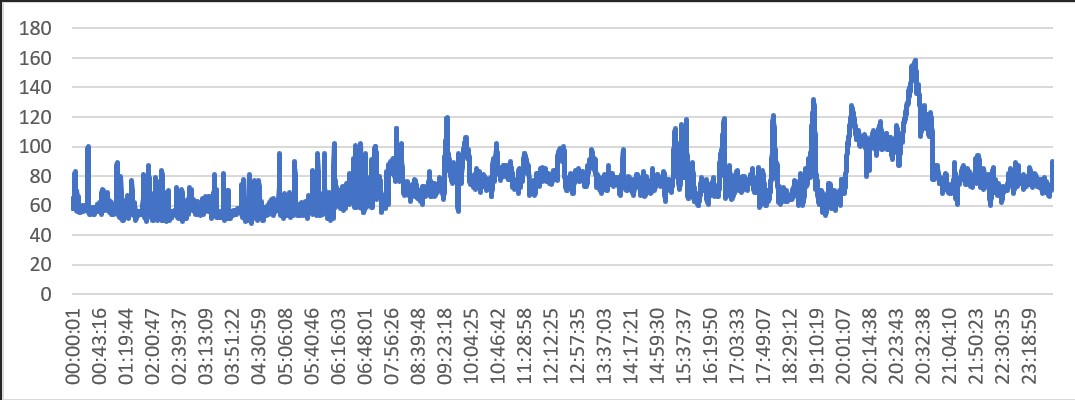

Supplement: Supplementary file 1 [file Image_1.jpeg]

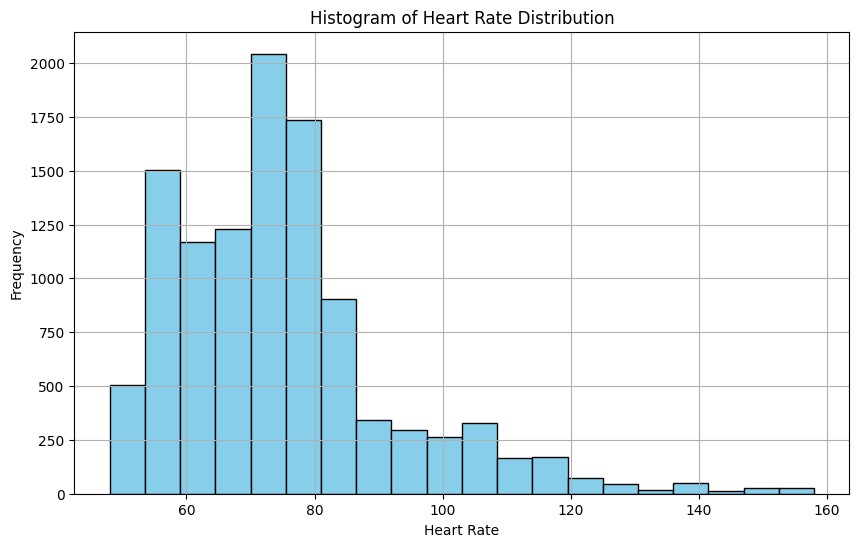

Supplement: Supplementary file 2 [file Image_2.jpeg]

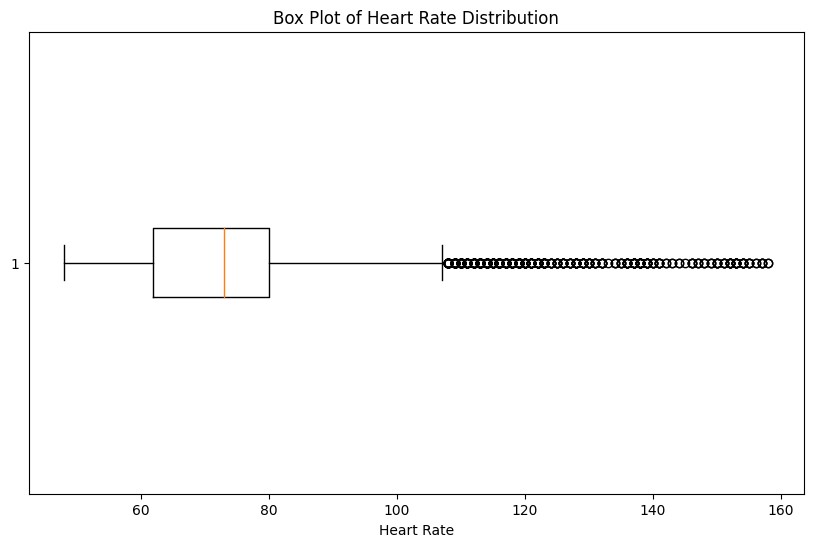

Supplement: Supplementary file 3 [file Image_3.jpeg]

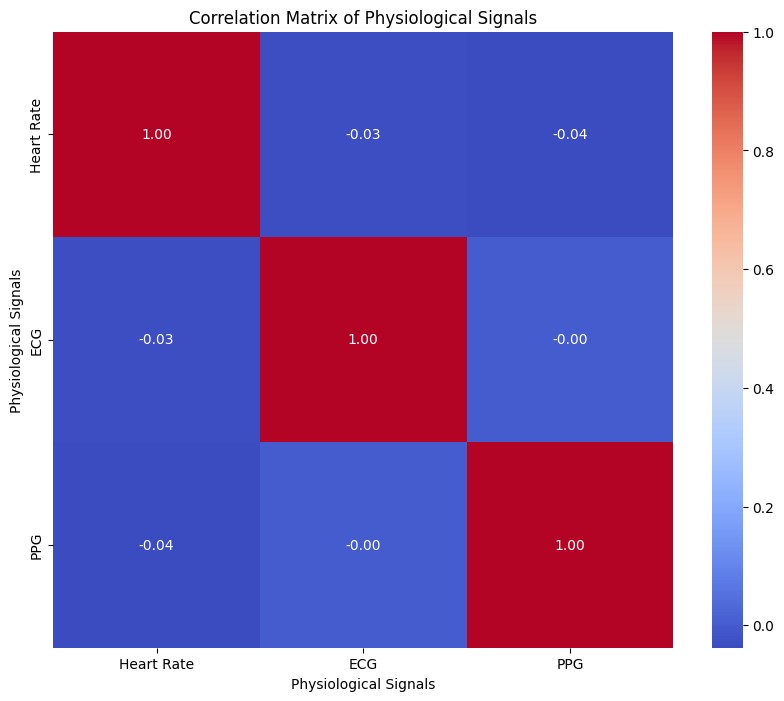

Supplement: Supplementary file 4 [file Image_4.jpeg]

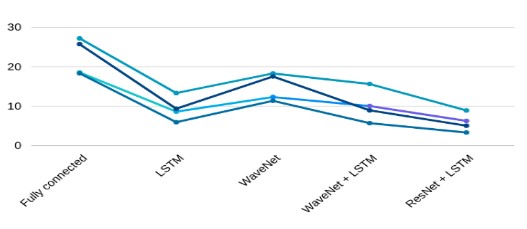

Supplement: Supplementary file 5 [file Image_5.jpeg]

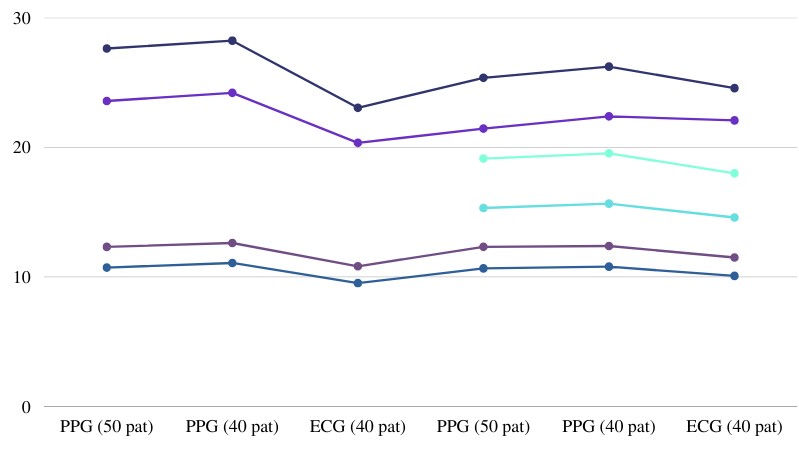

Supplement: Supplementary file 6 [file Image_6.jpeg]
